# Preparation and Corrosion Resistance Study of Nano-La_2_O_3_ Reinforced Electroless Ni-B Coatings

**DOI:** 10.3390/ma19122566

**Published:** 2026-06-13

**Authors:** Hongjie Li, Shaomu Wen, Yunqing Xia, Jizhong Yang, Chunyong Gu, Honglin Yang

**Affiliations:** 1 Research Institute of Natural Gas Technology, PetroChina Southwest Oil & Gasfield Company, Chengdu 610213, China; 18428301109@163.com (H.L.); 18380359324@163.com (Y.X.); 2 PetroChina Southwest Oil & Gasfield Company, Chengdu 610000, China; 3 PetroChina Southwest Oil and Gasfield Surface Engineering Design Center, PetroChina Southwest Oil & Gasfield Company, Chengdu 610213, China; 4 Central Sichuan Oil and Gas Mine, PetroChina Southwest Oil & Gasfield Company, Suining 629000, China

**Keywords:** electroless plating, Ni-B coating, nano-La_2_O_3_, anti-corrosion performance

## Abstract

**Highlights:**

- Fabricate Ni-B/La_2_O_3_ composite coatings by co-depositing nano-La_2_O_3_ particles into an electroless Ni-B alloy matrix.
- The optimal concentration was determined to be 8 g·L^−1^, evidenced by the best anti-corrosion property.
- The results conclusively show that nano-La_2_O_3_ reinforcement effectively enhances the corrosion resistance of electroless Ni-B alloy coatings.

**Abstract:**

This study was conducted to explore how varying the concentration of nano-La_2_O_3_ particles in the plating bath influences the morphology, constitution, and corrosion resistance of Ni-B composite coatings deposited on N80 carbon steel via electroless plating. The novelty of this work lies in the systematic investigation on the co-deposition behavior and grain refinement mechanism of nano-La_2_O_3_ in electroless Ni-B system, which has been rarely reported in previous studies. The microstructure and chemical composition of the coatings were characterized through a combination of SEM, EDS, XPS and XRD analyses. SEM confirmed that a dense Ni-B/La_2_O_3_ composite coating was formed, with a uniform thickness of approximately 10 μm, and the nano-La_2_O_3_ particles were evenly distributed. XPS analysis verified the presence of B, C, O, Ni and La, while XRD analysis revealed a refinement in crystalline size due to the addition of the nanoparticles. The corrosion resistance enhancement mechanism is attributed to the triple synergistic effect: nano-La_2_O_3_ pins grain boundaries and refines Ni-B grains to the minimum average size of 12.943 nm at the optimal concentration of 8 g·L^−1^; the refined grain structure promotes the formation of a continuous and dense Ni(OH)_2_ passive film; the uniformly dispersed nanoparticles act as physical barriers to block the penetration of corrosive media. Electrochemical measurements demonstrated that this coating exhibited outstanding anti-corrosion performance, as confirmed by a remarkably positive corrosion potential (*E_corr_* = −0.37189 V) and a minimal corrosion current density (*I_corr_* = 3.7524 μA/cm^2^). The results conclusively show that nano-La_2_O_3_ reinforcement effectively enhances the corrosion protection performance of electroless Ni-B alloy coatings.

## 1. Introduction

Metal corrosion poses a severe challenge to the industries, resulting in huge economic losses worldwide each year. The application of surface treatment technologies can not only relieve material degradation and maintain structural integrity, but also ensure the long-term stable operation of mechanical equipment by improving both corrosion and wear resistance. Among various surface modification technologies, electroless nickel–boron (Ni-B) alloy coatings have garnered significant interest in both academic and industrial circles over the past few decades [1,2], owing to their excellent weldability [3], hardness, wear [4] resistance, and chemical stability [5]. Electroless plating is based on a redox reaction in which metal ions in solution are reduced to metallic nickel by a strong reducing agent [6,7], yielding a coating that is both well-distributed and compact across the substrate surface [8,9]. This process is particularly suitable for surface treatment of components with complex geometries [10].

The boron content is the key factor in determining the microstructure and performance of Ni-B alloy coatings [11,12]. Coatings with low boron content typically exhibit a nano-crystalline structure, while increasing the boron content will cause the coating to gradually transition to an amorphous structure [13]. This evolution of structure is ascribed to lattice distortion and grain refinement caused by the solid solution of boron atoms in the nickel lattice [14,15]. Amorphous or nano-crystalline Ni-B alloys often demonstrate superior corrosion resistance owing to their significantly reduced grain-boundary density. However, engineering components in actual usage environments are often subjected to harsh conditions of corrosion and wear [16,17]. Under such circumstances, single-phase Ni-B coatings may fail to meet the comprehensive performance requirements for specific applications [18]. Consequently, the development of novel surface materials with structural and functional stability has become a major research hotspot in the area of surface engineering [19,20].

Composite coating technology, which involves the co-deposition of particles into a metal matrix [21], offers an effective approach to enhance the performance of coatings. Studies have shown that introducing micro- or nano-sized ceramic or rare-earth oxide particles (such as α-ZrP [22], SiC [12], Y_2_O_3_ [23], Si_3_N_4_ [24], MMT [25], Al_2_O_3_ [26], CeO_2_ [13]) into nickel-based alloy coatings can significantly improve their hardness, abrasive resistance, and anti-corrosion properties [26,27]. The strengthening mechanisms mainly include the dislocation pinning by the dispersed reinforcing particles [28], the filling of microscopic defects, leading to optimization of the coating microstructure, and the formation of a barrier that hinders the penetration of corrosive media [29]. In particular, rare-earth oxides exhibit considerable potential in metal-matrix composite coatings owing to their unique electronic structures and chemical activity.

Although techniques such as heat treatment can improve coating performance by promoting crystallization and the precipitation hardening, they often simultaneously intensify corrosion and wear, and may lead to uneven treatment of components with complex geometries. In contrast, composite co-deposition technology allows direct microstructural control during the plating process, offering a more promising route for the synergistic enhancement of properties. La_2_O_3_, as a typical rare-earth oxide, possesses excellent chemical stability, thermal resistance, and distinctive solubility properties under acidic conditions, and has been widely used in catalysis, ceramics and the electronics industry. While the strengthening effect of La_2_O_3_ in Ni-P, Ni-W and other alloy coatings has been reported, systematic studies on the co-deposition behavior of nano-sized La_2_O_3_ particles in electroless Ni-B systems and their synergistic impact on the microstructural characteristics and corrosion behavior of the coatings remain scarce. Given the importance of Ni-B-based composite coatings in surface engineering, a systematic investigation into the effect of La_2_O_3_ in modifying the microstructure and anti-corrosion properties of Ni-B matrix coatings carries clear scientific significance and engineering value.

This study aims to synthesize Ni-B/La_2_O_3_ composite coatings through co-depositing nano-La_2_O_3_ particles into an electroless Ni-B alloy matrix. The distribution characteristics of La_2_O_3_ nanoparticles in the coating and their influence on the phase constituents and grain refinement mechanism of Ni-B alloy matrix were mainly studied, thereby revealing the intrinsic correlation between the composite coating’s anti-corrosion properties and its microstructure. The clear novelty of this work is that it fills the key research gap: systematic studies on the co-deposition behavior, grain refinement mechanism and corrosion resistance enhancement mechanism of nano-La_2_O_3_ in electroless Ni-B systems are extremely scarce, while the strengthening effect of La_2_O_3_ has only been reported in Ni-P, Ni-W and other alloy systems. This work clarifies the synergistic strengthening mechanism of nano-La_2_O_3_ in electroless Ni-B coatings, including grain boundary pinning, grain refinement and passive film stabilization. The findings offer both theoretical insights and technical guidance for fabricating advanced Ni-B based composite coatings.

## 2. Materials and Methods

### 2.1. Materials and Reagents

N80 carbon steel specimens (30 mm × 15 mm × 3 mm) were utilized as substrates for electroless plating. The chemical reagents employed for coating preparation and their specifications are summarized in Table 1. All chemical reagents were of analytical grade and used directly as supplied without any further treatment. Nickel (*II*) sulfate hexahydrate [NiSO_4_·6H_2_O] and sodium acetate [CH_3_COONa] served as the nickel source and complexing agent, respectively. Dimethylamine borane [DMAB] functioned as the reducing agent, while thiourea [CH_4_N_2_S] was added as a stabilizer. Sodium dodecyl sulfate [SDS] served as an anionic surfactant to promote uniform dispersion of La_2_O_3_ nanoparticles in the plating bath. Nanoscale lanthanum (*III*) oxide (La_2_O_3_, 99.9%) having an average particle diameter of 40–80 nm (Shanghai Aladdin Biochemical Technology Co., Ltd., Shanghai, China) was used as the reinforcement phase. All other chemicals are purchased from Chengdu Kelong Chemical Co., Ltd. Sichuan, China.

### 2.2. Substrate Pretreatment

Prior to deposition, the N80 steel substrates underwent a multi-step surface treatment process to achieve a clean surface and promote strong coating adhesion. The details are as follows: (1) mechanical grinding was carried out using silicon carbide (SiC) grinding paper with particle sizes of 280#, 400# and 1000# in sequence to eliminate surface oxides and achieve a mirror-like smoothness; (2) ultrasonic treatment with acetone for 10 min to eliminate organic residues; (3) rinsing with deionized water (*DI*); (4) ethanol rinsing to remove any remaining water; (5) rinsing again with deionized water (*DI*); (6) immersing in a 10% volume hydrochloric acid solution for 15–20 s for surface activation at room temperature; (7) immediate DI water rinsing followed by rapid transfer to the plating bath. All rinsing steps were performed with DI water to minimize surface re-oxidation.

### 2.3. Plating Bath Formulation and Process Parameters

The electroless Ni–B/La_2_O_3_ composite plating bath was formulated based on previously optimized Ni–B alloy compositions. The detailed formulation of the plating bath and the key process parameters are provided in Table 1 and Table 2, respectively. The basic electrolyte contained 30 g·L^−1^ NiSO_4_·6H_2_O, 4.4 g·L^−1^ CH_3_COONa, 3 g·L^−1^ DMAB, 0.001 g·L^−1^ thiourea, and 0.00015 g·L^−1^ SDS. The plating process was conducted under controlled conditions: temperature 70 ± 1 °C, pH 6.0 ± 0.1, magnetic stirring rate 800 rpm, and deposition duration 45 min. pH adjustment was carried out with dilute sodium hydroxide or sulfuric acid solutions and monitored using a Five Easy Plus pH meter (Mettler-Toledo, Greifensee, Switzerland).

### 2.4. Fabrication of Electroless Ni–B/La_2_O_3_ Composite Coatings

To investigate the influence of La_2_O_3_ incorporation, varying amounts of nano-sized La_2_O_3_ particles (0, 4, 6, 8, and 10 g·L^−1^) were introduced into the base electrolyte. The nanoparticle dispersion was achieved through a three-stage sonication protocol: (*i*) initial ultrasonication at 40 kHz for 30 min using a KQ-100DE ultrasonic cleaner (Kunshan Ultrasonic Instrument Co., Ltd., Guangdong, China); (*ii*) subsequent probe sonication for 10 min to break nanoparticle agglomerates; and (*iii*) continuous magnetic stirring at 800 rpm during plating to prevent sedimentation.

The plating procedure involved transferring 200 mL of the prepared electrolyte into a 250 mL glass beaker, which was then placed in a DF-101S thermostatic magnetic stirrer (Shanghai Lichen Instrument Technology Co., Ltd., Shanghai, China) and warmed to the operating temperature. A PTFE-coated magnetic stir bar was employed to maintain uniform agitation. The pretreated N80 steel substrates were immediately immersed in the bath for the designated deposition period. Post-deposition, the coated specimens were quenched in cold water for 10 min, thoroughly rinsed with DI water to remove loosely adhered particles, and dried in a DHG-9070A electric drying oven (Shanghai Yiheng Scientific Instrument Co., Ltd., Shanghai, China) at 60 °C for 2 h.

### 2.5. Characterization

**Figure 1:**
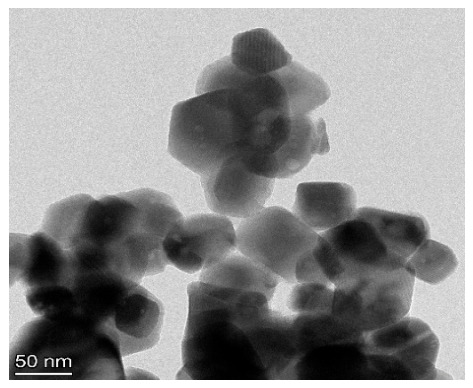
The TEM image of nano-La_2_O_3_ particles.

Before analyzing the coating, we observed the size of the nano-La_2_O_3_ particles and found that their size ranged from 30 to 100 nanometers, as shown in Figure 1.

Scanning electron microscopy (SEM) was employed to characterize the surface features and cross-sectional structure of the as-deposited coatings by using a Phenom ProX microscope (Phenom-World, Eindhoven, The Netherlands) equipped with energy disperse spectroscopy (EDS) for elemental composition analysis. The crystalline phases and structural characteristics were analyzed through X-ray diffraction (XRD) using an X’pert PRO diffractometer (PANalytical, Almelo, The Netherlands) with Cu Kα radiation source (*λ* = 0.15406 nm, 40 kV, 40 mA) within 20–90° with a 0.02° scanning step. X-ray photoelectron spectroscopy (XPS, Thermo Fisher Scientific, Waltham, MA, USA) employing monochromatic Al Kα radiation (*hν* = 1486.6 eV) was used to determine the chemical states of the elements. All XPS spectra were referenced to the adventitious C 1s peak (284.8 eV). It should be noted that energy-dispersive X-ray spectroscopy (EDS) has inherent limitations in accurately quantifying light elements such as boron (B) due to the low fluorescence yield of light elements and the strong spectral overlap between B-Kα (≈183 eV) and Ni-Mζ (≈186 eV) lines. Therefore, the B contents reported in Table 3 are semi-quantitative and serve primarily for comparative purposes rather than as absolute values.

Cross-sectional SEM analysis was employed to estimate the coating thickness at five random locations, with the average value reported. Electrochemical corrosion behavior was assessed using a CS2350 electrochemical workstation (Wuhan Corrtest Instruments Corp., Hubei, China) in a conventional three-electrode system. A saturated calomel electrode (SCE), a platinum plate, and the coated specimen (with a defined working area of 1 cm^2^) functioned as the reference, counter, and working electrodes, respectively. Potentiodynamic polarization curves were conducted in 3.5 wt.% NaCl solution (25 ± 2 °C, 1 mV·s^−1^) after 30 min open-circuit potential stabilization. Electrochemical impedance spectroscopy (EIS) was performed at the open-circuit potential by applying a 10 mV sinusoidal signal over a frequency range sweeping from 100 kHz to 10 mHz. Prior to electrochemical testing, all samples were ultrasonically cleaned in anhydrous ethanol for 1 min (to remove surface dust and trace amounts of loose oxide layers), then immediately dried with high-purity nitrogen, and finally quickly placed into the electrochemical cell for testing, ensuring that re-oxidation of the sample surfaces was prevented throughout the process. In addition, the active area of all samples was fixed at 1 cm^2^, and the encapsulation method and exposed surface conditions were identical. All electrochemical measurements were repeated at least three times using independently prepared coatings. The representative curves are shown, and the standard deviation for *E_corr_* and *I_corr_* is within ±5%.

## 3. Results

**Figure 2:**
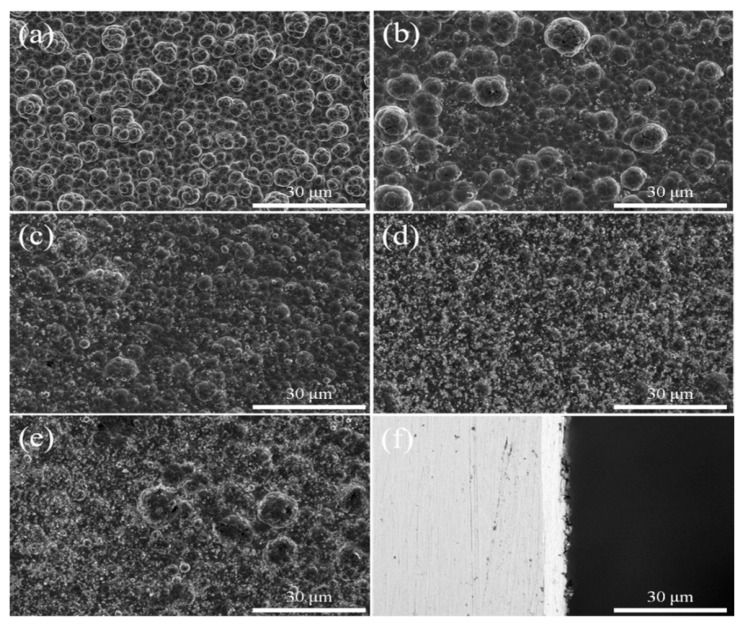
SEM micrographs of Ni-B/La_2_O_3_ composite coating with different La_2_O_3_ addition in the bath: (**a**) 0 g·L^−1^, (**b**) 4 g·L^−1^, (**c**) 6 g·L^−1^, (**d**) 8 g·L^−1^, (**e**) 10 g·L^−1^ and (**f**) coating/substrate interface of composite coating at the nano-La_2_O_3_ concentration (8 g·L^−1^).

The morphology of the Ni-B coating is displayed in Figure 2a, and the morphological characteristics of the Ni-B/La_2_O_3_ composite coating surface deposited under different concentrations of nano-La_2_O_3_ is shown in Figure 2b–e, and the loading of nano-La_2_O_3_ in the electroless plating bath ranges from 0 g·L^−1^ to 10 g·L^−1^. The magnification from Figure 2a–e is 5000×. It is evident from Figure 2a that the outer layer of Ni-B alloy coating has a densely arranged spherical structure. Observing Figure 2b–e, it is apparent that the surface of Ni-B/La_2_O_3_ composite coating does not have pinholes, cracks or other defects that affect the wholeness of the deposited layer, and the surface is compact and well-distributed. With the increase in nano-La_2_O_3_ particle content, a gradual increase in surface material is observed, accompanied by a change in surface morphology. This observation could be explained by the progressive enhancement of surface deposit coverage with increasing nano-La_2_O_3_ particle concentration in the plating bath. Among them, when the addition of nano-La_2_O_3_ reached 8 g·L^−1^ (Figure 2d), the composite coating demonstrates enhanced surface quality, with a more compact and homogeneous morphology.

However, when the addition of nano-La_2_O_3_ reached 4 g·L^−1^ (Figure 2b), the clustering of nano-La_2_O_3_ resulted in the development of distinct clusters on the composite coating surface. Excessive agglomeration of nano-La_2_O_3_ particles may lead them to fall off. This detachment, combined with the formation of large inter-nodular gaps, leads to an increase in surface defects on the coating, as shown in Figure 2e. In addition, hydrogen production can also promote defect formation on the coating surface during electroless plating at higher nanoparticle concentrations. These factors can lead to a deterioration in coating properties. Figure 2f illustrates the results of the Ni-B/La_2_O_3_ composite coating at the nano-La_2_O_3_ concentration (8 g·L^−1^) to investigate the coating/substrate interface, as well as the coating thickness. The thickness of composite coating is about 10.3 μm. The composite coating-metal matrix interface is free from structural imperfections, including micro-cracks, cracks, and delamination. This defect-free interface suggests a compact bonding and superior adhesion between the coating and the substrate.

**Figure 3:**
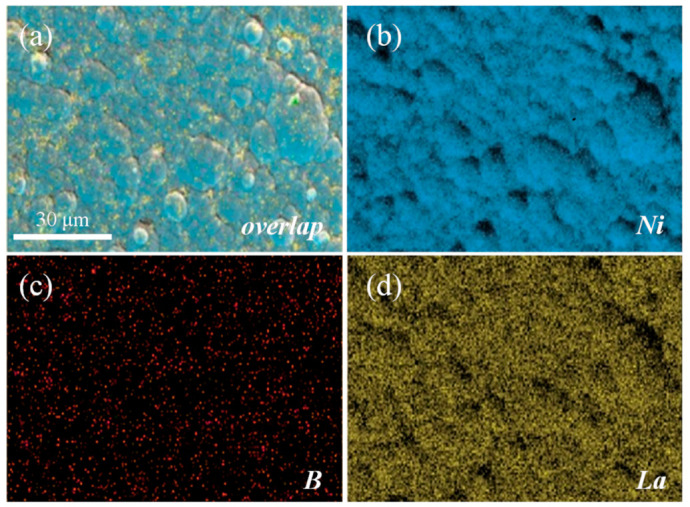
EDS mapping of the Ni-B/La_2_O_3_ composite coating (8 g·L^−1^): (**a**) overlap of elemental distribution, (**b**) Ni elemental distribution, (**c**) B elemental distribution, (**d**) La elemental distribution.

To better visualize the distribution of nano-La_2_O_3_ within the composite coating, we performed an EDS mapping on the surface region of Ni-B/La_2_O_3_ composite coating at the nano-La_2_O_3_ concentration (8 g·L^−1^) as shown in Figure 3. From the region selected in the scan, the coating is found to contain predominantly Ni, B, and La. A nano-La_2_O_3_ composite coating was successfully fabricated, as evidenced by the results. Additionally, it was found that the nano-La_2_O_3_ particles are homogeneously distributed throughout the coating. However, due to the relatively shallow scanning depth of the scanning electron microscope, the detected content of La in the coating is low. As B is a light element, its signal may be overshadowed by that of Ni, leading to an underestimation of its content. Table 3 provides a detailed EDS element distribution of Ni-B/La_2_O_3_ composite coating. The B content is 1.20 wt.%, which is basically consistent with Ni-B coating prepared using DMAB as the boron precursor, which typically contain 0.1–2 wt.% B.

**Figure 4:**
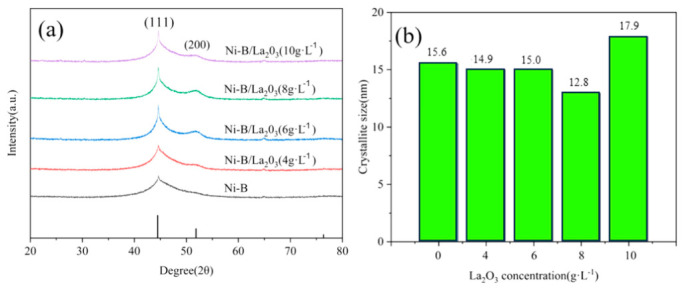
The XRD pattern (**a**) and crystalline size (**b**) of Ni-B/La_2_O_3_ composite coating.

In order to gain insight into the performance-related crystallographic features, the Ni-B/La_2_O_3_ composite coating was characterized by XRD to determine its crystal planes and crystallite size. The obtained diffraction patterns are presented in Figure 4. In Figure 4a, we found Ni characteristic peaks at 44.496° and 51.849°, assigned to the Ni (111) and Ni (200), respectively. The Ni-B/La_2_O_3_ composite coatings exhibit broad and poorly defined Ni diffraction peaks, which are characteristic of nanocrystalline or partially amorphous structures. The significant peak broadening arises from two main factors: (*i*) the fine crystallite size (12–20 nm, as calculated in Figure 4b) and (*ii*) lattice microstrain induced by the solid solution of boron atoms into the nickel lattice. Notably, only the Ni (111) peak is clearly distinguished, while the Ni (200) peak appears much weaker and broader. This phenomenon is commonly observed in nanocrystalline Ni-based coatings and can be explained by a preferred (111) crystallographic orientation and significant anisotropic microstrain, which preferentially broadens the (200) reflection.

At the same time, the incorporation of nano-La_2_O_3_ led to changes in the relative peak intensities; however, this had no effect on the crystalline phase formation. Upon increasing the nano-La_2_O_3_ content in the electrolyte (0 g·L^−1^ to 4 g·L^−1^), a reduction in the relative peak intensities was observed, emphasizing that the coating has smaller crystalline sizes. This may be due to the pinning effect of nano-La_2_O_3_, which suppresses the movement of grain boundaries, thus inhibiting grain growth and reducing the crystalline size of the coating. When the nano-La_2_O_3_ content reached 6 g·L^−1^ and 10 g·L^−1^, the Ni (111) peak became more intense and its shape sharpened considerably. This may be attributed to agglomeration, which reduces nucleation sites and consequently increases crystallite size. Furthermore, no characteristic diffraction peaks of nano-La_2_O_3_ were observed in the XRD patterns, this phenomenon possibly arising from the fact that the amount of nano-La_2_O_3_ present at the composite coating surface falls below the detection threshold of XRD.

The average crystalline size is used to further verify the function of nano-La_2_O_3_ on the coating crystalline size. The crystallite size was calculated using Scherrer’s equation:

(1) $$D=\frac{K\gamma }{\mathrm{Bcos}\theta }$$

In Scherrer’s equation, *D* corresponds to the mean crystallite size, *K* denotes the Scherrer constant (typically 0.89), *λ* represents the X-ray wavelength (1.54056 Å), *β* represents the peak broadening at half maximum intensity (FWHM), and *θ* corresponds to the Bragg angle. The peak broadening (FWHM) values were obtained by fitting the Ni (111) and Ni (200) diffraction peaks using a pseudo-Voigt function after background subtraction in X’Pert HighScore Plus software 2.2a. The instrumental broadening was calibrated using a standard LaB_6_ sample. The corrected FWHM was then used in the Scherrer equation. Figure 4b demonstrates that the average grain size of the Ni-B/La_2_O_3_ composite coating decreases up to a certain nano-La_2_O_3_ concentration, beyond which it increases. The Ni-B/La_2_O_3_ (8 g·L^−1^) composite coating has the minimum grain size (12.943 nm). However, adding excessive nano-La_2_O_3_ to the deposition solution leads to increased accumulation, and the agglomeration of nanoparticles hinders the development of the coating’s surface microstructure. This further confirms that uniformly dispersed nano-La_2_O_3_ is beneficial for grain refinement of the coating, forming a relatively dense microstructure, which is one of the factors responsible for the enhanced coating performance.

**Figure 5:**
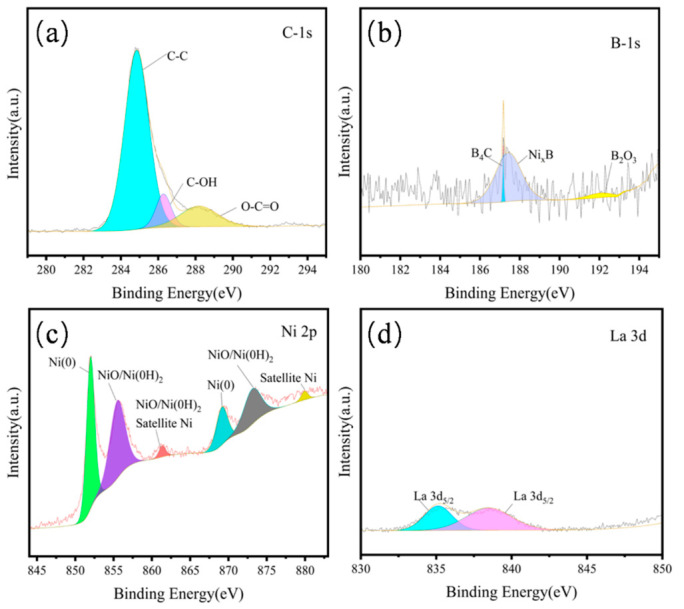
XPS spectra of the Ni-B/La_2_O_3_ composite coating: (**a**) high-resolution C 1s spectrum, (**b**) high-resolution B 1s spectrum, (**c**) high-resolution Ni 2p spectrum and (**d**) high-resolution La 3d spectrum.

To further demonstrate that nano-La_2_O_3_ has been successfully mixed into the Ni-B coating, we analyzed the bond energy of the compound using XPS as displayed in Figure 5, which demonstrates the XPS test diagram of the Ni-B/La_2_O_3_ composite coating, which contains four elements: C, B, Ni and La. It should be noted that energy-dispersive X-ray spectroscopy (EDS) has inherent limitations in accurately quantifying light elements such as boron (B) due to the low fluorescence yield of light elements and the strong spectral overlap between B-Kα (≈183 eV) and Ni-Mζ (≈186 eV) lines. Therefore, the B contents reported in Table 3 are semi-quantitative and serve primarily for comparative purposes rather than as absolute values. As shown in Figure 5a, the high-resolution C 1s spectrum can be fitted with three components centered at binding energies of 284.83, 286.28, and 288.18 eV, which are attributed to C–C, C–OH, and O–C=O species, respectively. Figure 5b shows the 1s spectra of element B, with peaks at 187.18, 187.43 and 192.13 eV, which can be assigned to B_4_C, Ni_X_B and boron oxide, respectively. As presented in Figure 5c, the high-resolution Ni 2p spectrum can be resolved into three components. The peaks at 852.03, 855.53, and 861.38 eV correspond to metallic Ni (Ni0), NiO/Ni(OH)_2_ species, and the corresponding shake-up satellite, respectively, all of which belong to Ni 2p3/2. Meanwhile, the features observed at 869.18, 873.28, and 879.93 eV are attributed to metallic Ni (Ni0), NiO/Ni(OH)_2_ species, and the corresponding shake-up satellite, respectively, which belongs to Ni 2p1/2. The presence of Ni(OH)_2_ suggests that metallic Ni on the Ni-B/La_2_O_3_ composite coating surface has undergone oxidation, forming a nickel oxide film. Studies have shown that this nickel oxide film is preferentially formed as a passivation product at the grain boundary and forms an adherent barrier layer on the coating surface, thereby enhancing corrosion protection. Figure 5d shows the 3d spectra of the element La, where the La 3d spectrum displays two distinct peaks at 835.13 eV and 838.48 eV, corresponding to the La 3d_5/2_ core level. The presence of these characteristic La signals verifies that nano-La_2_O_3_ has been successfully embedded within the Ni-B coating, confirming the successful fabrication of the Ni-B/La_2_O_3_ nanocomposite.

**Figure 6:**
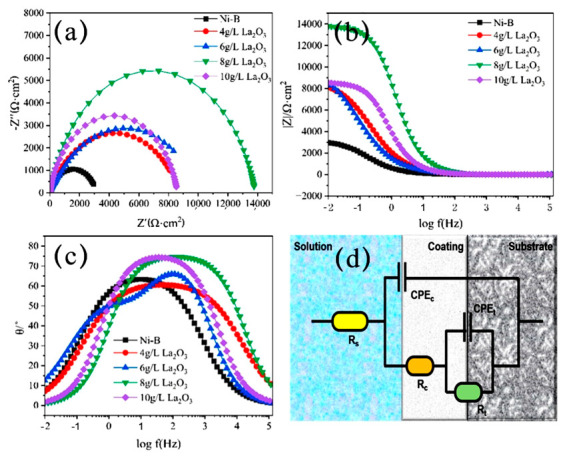
EIS analyses of Ni-B/La_2_O_3_ composite coatings: (**a**) Nyquist plots, (**b**) Bode plots, (**c**) phase angle plots, (**d**) Randles equivalent circuit model.

Figure 6 shows the EIS results. It is obvious that the impedance of all Ni-B/La_2_O_3_ composite coatings with added nano-La_2_O_3_ surpass that of the unmodified Ni-B coating (Figure 6a), indicating that incorporation of nano-La_2_O_3_ effectively enhances the coating’s corrosion resistance. Among them, the composite coating containing 8 g·L^−1^ nano-La_2_O_3_ displays the greatest impedance semicircle diameter, signifying superior corrosion protection. The rest are ranked in descending order of impedance as Ni-B/La_2_O_3_ (10 g·L^−1^) composite coating, Ni-B/La_2_O_3_ (6 g·L^−1^) composite coating, Ni-B/La_2_O_3_ (4 g·L^−1^) composite coating, and Ni-B coating. Figure 6b is the Bode plot of the Ni-B/La_2_O_3_ (0 g·L^−1^–10 g·L^−1^) composite coatings. The results show that in the low-frequency region, the Ni-B/La_2_O_3_ composite coatings exhibit excellent performance and maintain a prominent trend. Furthermore, the impedance modulus (|*Z*|) at 0.01 Hz for each Ni-B/La_2_O_3_ composite coating is consistently higher than the unmodified Ni-B coating, and the |*Z*| value at 0.01 Hz of the Ni-B/La_2_O_3_ (8 g·L^−1^) composite coating is the highest. This is consistent with the impedance plot results, providing additional evidence that incorporating nano-La_2_O_3_ into the coating leads to improved corrosion protection, and among all formulations, the composite coating containing 8 g·L^−1^ nano-La_2_O_3_ demonstrates the highest corrosion protection.

As revealed in Figure 6c, the phase angle response of the Ni-B/La_2_O_3_ coatings varies with La_2_O_3_ content. The broadest and most elevated phase angle peak is observed at 8 g·L^−1^, signifying the most compact and uniform surface structure, which correlates well with the enhanced corrosion resistance and the SEM microstructural findings.

In the case of a single time constant response, the widely used Randles equivalent circuit (Figure 6d) is used for quantitative analysis. An equivalent circuit model is used for fitting the EIS data: *R_s_*(*CPE_c_*(*R_c_*(*CPE_t_R_t_*))).

*R_c_* and *CPE_c_* represent the resistance and constant phase element of the outer porous coating layer, and *R_t_* and *CPE_t_* correspond to the inner barrier layer charge transfer process, which represents the resistance and constant phase element of the inner coating layer, the data of which are shown in Table 4. Pitting corrosion in mild steel is mainly attributed to oxygen and chloride ions. Therefore, corrosion resistance can be substantially improved by introducing agents that either impede the transport of these ions or decrease the chloride ion content via chemical reactions. Within the *R_s_*(*CPE_c_*(*R_c_*(*CPE_t_R_t_*))) equivalent circuit, *CPE_c_* corresponds to the double-layer capacitance at the electrode–electrolyte interface, which rises as the electrolyte permeates toward the substrate. Therefore, starting from the value of *CPE_c_*, we can also analyze the erosion effect of the electrolyte solution on the coating. In the table, the data confirm that the coating with 8 g·L^−1^ La_2_O_3_ exhibits the lowest *CPE_c_*; it is suggested that the adsorption of chloride ions by the electrical double layer is significantly reduced by the presence of nanoparticles, making it more difficult for these ions to reach the coating surface and thus lowering the corrosion rate. Meanwhile the data confirm that the coating with 8 g·L^−1^ La_2_O_3_ exhibits the highest inner layer resistance, consistent with its superior corrosion resistance.

To quantify and verify the rationality of the selected equivalent circuit, we have supplemented the chi-square goodness of fit values (*χ*^2^) for each sample. The smaller the chi-square value, the higher the consistency between the fitting results and the experimental data. Generally, for EIS fitting, if the *χ*^2^ value is on the order of 10^−3^, the fitting results are considered reliable. As shown in Table 4, the fitted chi-square values of all samples are at relatively low levels, indicating that fitting this composite coating system with a single-time-constant Randles circuit not only meets the requirements of physical model simplification but also has high mathematical fitting accuracy.

**Figure 7:**
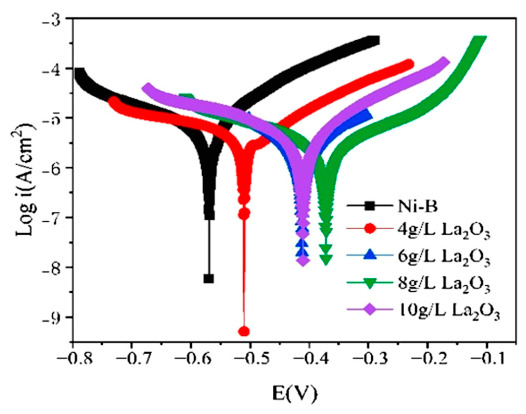
The polarization curves of Ni-B/La_2_O_3_ composite coating.

Figure 7 illustrates the polarization curves of Ni-B/La_2_O_3_ composite coating. The dynamic potential polarization was performed at a scan rate of 1 mV/s, from cathode to anode (from OCP − 200 mV to OCP + 200 mV). Based on these curves, the Tafel parameters (*β_a_*, *β_c_*, *E_corr_*, *I_corr_*) were obtained by Tafel extrapolation, as shown in Table 5. In polarization curve analysis, the corrosion potential (*E_corr_*) is the open-circuit potential of the metal measured without external current. *E_corr_* serves as an indicator of corrosion susceptibility: a higher (more noble) *E_corr_* value typically corresponds to better corrosion resistance, whereas a lower (more active) *E_corr_* indicates a greater tendency for the material to lose electrons and undergo oxidation, thereby increasing its corrosion susceptibility. From the polarization curves, it is apparent that the addition of nano-La_2_O_3_ shifts the Ni-B/La_2_O_3_ composite coating’s polarization curve to more positive corrosion potential values. Table 5 also shows that with increasing nano-La_2_O_3_ concentration in the Ni-B/La_2_O_3_ composite coating, *E_corr_* displaces to more positive potentials, while *I_corr_* decreases. This suggests that the coating exhibits enhanced inertness and superior corrosion resistance. Meanwhile, the Ni-B/La_2_O_3_ (8 g·L^−1^) composite coating achieves the most positive *E_corr_* (*E_corr_* = −0.37189 V) coupled with the minimum *I_corr_* (*I_corr_* = 3.7524 μA/cm^2^). Upon further increasing the nano-La_2_O_3_ content, the *E_corr_* of the composite coating shifts to more negative values, accompanied by an increase in *I_corr_*, implying a reduction in the coating’s inertness and a decline in corrosion resistance. The more positive corrosion potential coupled with low corrosion current sufficiently demonstrate that the Ni-B/La_2_O_3_ (8 g·L^−1^) composite coating has optimal corrosion resistance.

## 4. Discussion

This study used electroless plating technology to prepare a highly corrosion-resistant Ni-B/La_2_O_3_ composite coating and conducted a detailed investigation into its morphology, structure, composition, and corrosion resistance, as well as the influence of varying nano-La_2_O_3_ particles concentrations (ranging from 4 g·L^−1^ to 10 g·L^−1^) on the corrosion protection ability of the coating.

### 4.1. Mechanism of La_2_O_3_ in Grain Boundary Pinning and Grain Refinement

The observed grain refinement in the Ni-B/La_2_O_3_ composite coatings can be primarily attributed to the heterogeneous nucleation effect and the Zener pinning mechanism induced by the dispersed La_2_O_3_ nanoparticles. During the initial stages of electroless deposition, the uniformly dispersed nano-La_2_O_3_ particles act as preferential nucleation sites for Ni and B atoms. This increases the nucleation density, which is critical for the formation of a fine-grained structure. Concurrently, the nanoparticles embedded within the growing matrix interact with grain boundaries, exerting a pinning force that impedes their migration. According to the Zener pinning principle, the presence of second-phase particles restricts grain boundary movement, thereby inhibiting grain growth and stabilizing the fine-grained microstructure. The XRD analysis corroborates this mechanism, showing that the average crystallite size of the Ni-B matrix decreases progressively with increasing La_2_O_3_ content up to 8 g·L^−1^ (from 15.6 nm for the plain Ni-B coating to 12.8 nm for the Ni-B/La_2_O_3_ (8 g·L^−1^) composite coating). This reduction in crystallite size leads to a higher density of grain boundaries, which can contribute to the formation of a more uniform and compact passive film. However, at an excessive concentration of 10 g·L^−1^, the crystallite size increases again to 17.9 nm, likely due to the agglomeration of nanoparticles, which reduces the number of effective nucleation sites and weakens the pinning effect.

### 4.2. Contribution of the Ni(OH)_2_ Passive Film to Corrosion Resistance at Optimal Concentration

The XPS analysis revealed the presence of Ni(OH)_2_ species on the surface of the Ni-B/La_2_O_3_ composite coating. The formation of this nickel hydroxide layer is a key factor in the enhanced corrosion resistance. In the corrosive NaCl electrolyte, the nickel matrix undergoes an initial oxidation, forming a thin, adherent, and stable Ni(OH)_2_ passive film. This layer acts as a physical barrier that effectively separates the underlying coating from the corrosive environment, hindering the ingress of aggressive Cl^−^ ions and the egress of metal cations.

At the optimal La_2_O_3_ concentration of 8 g·L^−1^, the synergistic effect of grain refinement and passive film formation is maximized. The significantly reduced crystallite size (12.8 nm) provides a high density of grain boundaries, which act as rapid diffusion paths for the formation of a continuous and uniform passive layer. The uniformly distributed La_2_O_3_ nanoparticles also serve as inert fillers within the passive film, enhancing its compactness and stability. This results in a more effective barrier with fewer defects, which is reflected in the EIS data. The Ni-B/La_2_O_3_ (8 g·L^−1^) coating exhibits the highest charge transfer resistance (*R_t_* = 13,780 Ω·cm^2^) and the lowest constant phase element (*CPE*) value, indicating a highly stable and protective electrode/electrolyte interface with reduced ionic diffusion. The passive film formed at this concentration is more resistant to localized breakdown, leading to the observed noble corrosion potential (*E_corr_* = −0.37189 V) and minimal corrosion current density (*I_corr_* = 3.7524 μA/cm^2^).

To further substantiate the relationship between microstructure (grain refinement) and electrochemical behavior (corrosion resistance), we performed a quantitative analysis of the crystallite size, charge transfer resistance (*R_t_*) and corrosion current density (*I_corr_*) for all coatings. The data are summarized in Table 6.

To quantitatively assess the correlation between grain size and corrosion resistance, the Pearson correlation coefficient (*r*) was calculated using the following formula:

$$r=\frac{\sum_{i=1}^{n}\left(x_{i}-\bar{x})(y_{i}-\bar{y}\right)}{\sqrt{\sum_{i=1}^{n}{\left(x_{i}-\bar{x}\right)}^{2}}\sqrt{\sum_{i=1}^{n}{\left(y_{i}-\bar{y}\right)}^{2}}}$$

where *x_i_* represents the crystallite size of each coating, *y_i_* represents the corresponding *R_t_* (or *I_corr_*), and $\bar{x}$ and $\bar{y}$ are the mean values of crystallite size and the electrochemical parameter, respectively. The calculations were performed using the data in Table 6.

The Pearson correlation coefficient between crystallite size and *R_t_* was found to be *r* = −0.54, indicating a moderate negative correlation. This implies that coatings with smaller crystallite sizes tend to exhibit higher charge transfer resistance, i.e., superior corrosion protection.

The Pearson correlation coefficient between crystallite size and *I_corr_* was *r* = +0.41, showing a weak to moderate positive correlation. This suggests that a reduction in grain size is generally accompanied by a lower corrosion current density.

These statistical results confirm that grain refinement, achieved by optimal incorporation of nano-La_2_O_3_ (8 g·L^−1^), directly contributes to enhanced corrosion resistance. The moderate negative correlation with *R_t_* and the positive correlation with *I_corr_* support the mechanistic interpretation that finer grains promote the formation of a more uniform and stable passive film (Ni(OH)_2_), as discussed in Section 4.1 and Section 4.2. Although the correlation coefficients are not extremely high due to the limited sample size and the influence of other factors (e.g., nanoparticle distribution, porosity), the overall trend is consistent with the experimental observations and further validates the synergistic strengthening effect of nano-La_2_O_3_ in electroless Ni-B coatings.

### 4.3. Quantitative Comparison with Similar Rare-Earth Oxide Reinforced Ni-B Systems

To further validate the effectiveness of La_2_O_3_ as a reinforcement, the performance of the Ni-B/La_2_O_3_ (8 g·L^−1^) coating was compared quantitatively with other rare-earth oxide and layered material reinforced Ni-B systems reported in the literature, specifically Ni-B/CeO_2_ [13] and Ni-B/α-ZrP [22]. The key comparative parameters are summarized in Table 7.

The enhancement mechanism—grain refinement, passive film stabilization, and the creation of a more compact microstructure—is consistent with the findings for other rare-earth oxide reinforced systems. The results confirm that nano-La_2_O_3_ is an effective reinforcement for improving the anti-corrosion properties of electroless Ni-B coatings, although further optimization of dispersion and concentration could potentially yield even greater improvements comparable to the best-performing systems like Ni-B/α-ZrP.

In summary, the enhanced corrosion resistance of the Ni-B/La_2_O_3_ (8 g·L^−1^) composite coating is a result of a synergistic mechanism. The La_2_O_3_ nanoparticles promote grain refinement via heterogeneous nucleation and Zener pinning. This refined structure, in turn, facilitates the formation of a more uniform and stable Ni(OH)_2_ passive film. The embedded nanoparticles also act as inert barriers within the coating and the passive layer, increasing the tortuosity of the diffusion path for corrosive species and enhancing the overall integrity and protectiveness of the coating system. The slight decrease in performance at 10 g·L^−1^ is attributed to nanoparticle agglomeration, which introduces defects and compromises the uniformity of the coating and its passive film.

## 5. Conclusions

SEM observations manifested that the prepared Ni-B/La_2_O_3_ composite coating had good integrity, with no pinholes or microcracks, and was dense. Cross-sectional SEM analysis revealed a coating thickness of approximately 10 μm. nano-La_2_O_3_ particles were uniformly distributed in the coating. XRD and XPS results indicated that the coating was successfully modified with nano-La_2_O_3_ nanoparticles. Furthermore, the appropriate addition of nano-La_2_O_3_ refined the average grain size of the coating. The optimal concentration of nano-La_2_O_3_ was determined to be 8 g·L^−1^, at which the composite coating had the smallest average grain size of 12.943 nm.

Electrochemical experiments revealed that the Ni-B/La_2_O_3_ (8 g·L^−1^) composite coating exhibited superior corrosion protection, corresponding to the most noble corrosion potential (*E_corr_* = −0.37189 V) and the lowest corrosion current density (*I_corr_* = 3.7524 μA/cm^2^). Therefore, it can thus be concluded that nano-La_2_O_3_ particles act as an effective reinforcement, enhancing the anti-corrosion properties of Ni-B alloy coatings.

In summary, this study systematically elucidates the synergistic role of nano-La_2_O_3_ particles in tailoring the microstructure and corrosion resistance of electroless Ni-B composite coatings. By establishing a direct correlation between the concentration of nano-La_2_O_3_ in the plating bath and its incorporation into the coating matrix, we provide compelling evidence for a co-deposition mechanism that governs grain refinement and surface densification. The non-monotonic variation in grain size, initially decreasing with nano-La_2_O_3_ addition and then increasing at higher concentrations, reveals a critical threshold at 8 g·L^−1^, where optimal dispersion of nanoparticles leads to maximum grain refinement (12.8 nm) and superior corrosion protection (*E_corr_* = −0.37189 V, *I_corr_* = 3.7524 μA/cm^2^). Beyond this point, agglomeration of nanoparticles disrupts microstructural uniformity and compromises performance. These findings not only validate the effectiveness of nano-La_2_O_3_ as a reinforcement phase but also underscore the importance of precise concentration control in composite electrodeposition processes. The insights gained here offer a rational framework for designing high-performance Ni-B-based composite coatings with tunable microstructures and enhanced durability, with promising applications in corrosive environments such as those encountered in oil and gas industries.

## Figures and Tables

**Table 1:** Basic composition of the deposition bath for electroless Ni–B/La_2_O_3_ composite coatings.

Basic Electrolyte Composition	Concentration (g·L^−1^)
NiSO_4_·6H_2_O	30
CH_3_COONa	4.4
DMAB	3
CH_4_N_2_S	0.001
SDS	0.00015

**Table 2:** Process parameters for electroless Ni–B/La_2_O_3_ composite coatings.

Electroless Plating Parameters	Quantity
Stirring Rate	800 rpm
Time	45 min
Temperature	69–71°C
pH	5.9–6.1

**Table 3:** The element distribution of Ni-B/La_2_O_3_ composite coating.

Samples	B wt.%	Ni wt.%	La wt.%	B At%	Ni At%	La At%
Ni-B	1.6	98.4	-	7.6	92.4	-
Ni-B/La_2_O_3_ (4 g·L^−1^)	1.5	98.3	0.2	7.7	92.2	0.1
Ni-B/La_2_O_3_ (6 g·L^−1^)	1.3	98.2	0.5	7.6	92.2	0.2
Ni-B/La_2_O_3_ (8 g·L^−1^)	1.3	97.6	1.1	7.4	92.0	0.6
Ni-B/La_2_O_3_ (10 g·L^−1^)	1.0	97.6	1.1	7.4	91.9	0.7

**Table 4:** The EIS test data of Ni-B/La_2_O_3_ composite coating.

Samples	*R_s_* (Ω·cm^2^)	*CPE_c_ Y*_0_ (×10^−5^ Ω^−1^·cm^−2^·s^n^)	*n*_c_	*R_c_* (Ω·cm^2^)	*CPE_t_ Y*_0_ (×10^−5^ Ω^−1^·cm^−2^·s^n^)	*n_t_*	*R_t_* (Ω·cm^2^)	*Χ* ^2^
Ni-B	4.38	28.5	0.72	215	15.2	0.68	3100	5.2 × 10^−4^
Ni-B/La_2_O_3_ (4 g·L^−1^)	3.06	9.80	0.68	410	5.20	0.72	8620	6.1 × 10^−4^
Ni-B/La_2_O_3_ (6 g·L^−1^)	6.63	11.2	0.70	388	6.10	0.70	8510	4.8 × 10^−5^
Ni-B/La_2_O_3_ (8 g·L^−1^)	3.28	1.21	0.82	162	0.85	0.85	13780	5.6 × 10^−5^
Ni-B/La_2_O_3_ (10 g·L^−1^)	5.85	3.20	0.75	245	1.95	0.80	8490	3.2 × 10^−4^

**Table 5:** The polarization curves data of Ni-B/La_2_O_3_ composite coating.

Samples	*β_a_* (mV/decade)	*β_c_* (mV/decade)	*E_corr_* (V)	*I_corr_* (μA/cm^2^)
Ni-B	0.17329	0.44106	−0.56945	11.819
Ni-B/La_2_O_3_ (4 g·L^−1^)	0.25594	0.72152	−0.51016	8.6357
Ni-B/La_2_O_3_ (6 g·L^−1^)	0.39019	0.48088	−0.41212	9.0850
Ni-B/La_2_O_3_ (8 g·L^−1^)	0.22809	0.31475	−0.37189	3.7524
Ni-B/La_2_O_3_ (10 g·L^−1^)	0.19622	0.42062	−0.41075	7.0704

**Table 6:** Crystallite size, *R_t_* and *I_corr_* of Ni-B/La_2_O_3_ composite coatings.

Samples	Crystallite Size (nm)	*R_t_* (Ω·cm^2^)	*I_corr_* (μA/cm^2^)
Ni-B	15.6	3100	11.819
Ni-B/La_2_O_3_ (4 g·L^−1^)	14.9	8620	8.6357
Ni-B/La_2_O_3_ (6 g·L^−1^)	15.0	8510	9.0850
Ni-B/La_2_O_3_ (8 g·L^−1^)	12.8	13780	3.7524
Ni-B/La_2_O_3_ (10 g·L^−1^)	17.9	8490	7.0704

**Table 7:** Comparative performance of Ni-B matrix composite coatings reinforced with different nanoparticles.

Coating System	Optimal Particle Concentration (g·L^−1^)	Crystallite Size (nm)	*I_corr_* (μA/cm^2^) in 3.5 wt.% NaCl	*R_t_* (Ω·cm^2^)
Ni-B/La_2_O_3_	8	12.8	3.75	13780
Ni-B/CeO_2_ [13]	10	17.8	-	-
Ni-B/α-ZrP [22]	1.5	5.8	2.93	24870

## Data Availability

The original contributions presented in this study are included in the article. Further inquiries can be directed to the corresponding author.
